# The Impact of Highly Effective CFTR Modulators on Growth and Nutrition Status

**DOI:** 10.3390/nu13092907

**Published:** 2021-08-24

**Authors:** Rosara Bass, Jefferson N. Brownell, Virginia A. Stallings

**Affiliations:** 1 Children’s Hospital of Philadelphia, 3401 Civic Center Blvd, Philadelphia, PA 19104, USA; 2 School of Medicine, University of Pennsylvania Perelman, Perelman School of Medicine at the University of Pennsylvania, Philadelphia, PA 19104, USA; brownellj@chop.edu (J.N.B.); stallingsv@chop.edu (V.A.S.)

**Keywords:** CFTR modulator, CFTR corrector, nutrition

## Abstract

Patients with cystic fibrosis (CF) are at increased risk of malnutrition and growth failure due to multiple factors as a result of suboptimal or absent function of the CFTR chloride channel protein. Dysfunctional CFTR contributes to increased energy expenditure, exocrine pancreatic insufficiency causing impaired dietary macronutrient digestion and absorption, intestinal dysbiosis, and impaired bile acid homeostasis. Poor nutritional status as a result of these mechanisms is associated with decreased lung function, worse clinical outcomes, and ultimately, increased mortality. Nutritional interventions addressing these mechanisms, such as pancreatic enzyme-replacement therapy and enteral caloric supplementation, have improved nutritional status and, by association, clinical outcomes. In the last decade, the advent of medications targeting defective CFTR proteins has revolutionized the care of patients with CF by reducing the overall impact of CFTR dysfunction. Below, we summarize the effects of highly effective CFTR modulators on nutritional status overall as well as specific factors including bile acid metabolism, pancreatic function, energy expenditure, and intestinal dysbiosis. The future of CF nutrition care will require a paradigm shift away from focusing on methods addressing CFTR dysfunction such as excess calorie provision and toward an individualized, holistic approach in the context of specific mutations and CFTR-directed therapy.

## 1. CFTR Mutations and Cystic Fibrosis

The Cystic Fibrosis Transmembrane Conductance Regulator (CFTR) protein is an epithelial ion channel responsible for chloride transport across cell membranes. Mutations in the CFTR gene, which encodes for this protein, cause the disease cystic fibrosis (CF). Mutations in CFTR are classified from I to VI based on their functional effects [1,2,3]. To date, more than 2000 mutations have been found in the CFTR gene with variable effects on its function [4]. The most common CFTR mutation in the United States is the deletion of phenylalanine in position 508 (F508del), with 85% of individuals with CF possessing at least one copy of this mutation. F508del is a class II mutation, characterized by defective protein processing. While CFTR allele distributions vary highly among populations, no other mutation is currently found in more than five percent of individuals with CF in the United States. Based on data collected in the CF Patient registry, the next two most common mutations are G542X, a class I mutation in which no functional CFTR is created, and G551D, a class III mutation, characterized by defective protein regulation or gating. G542X and G551D are present in 4.5 and 4.4 percent of people with CF, respectively [3,4,5].

## 2. CFTR Modulators and Cystic Fibrosis

Until 2012 and the advent of CFTR modulators, therapies for CF targeted secondary manifestations of CFTR dysfunction. CFTR modulators are the first therapies to target the primary mechanism of CFTR protein dysfunction [5]. Currently available modulators are classified as either potentiators, which affect the channel opening, or “gating” properties of mutant CFTR, or correctors, which stabilize mutant CFTR protein early in biogenesis [5,6].

Ivacaftor (VX-770; Kalydeco, Vertex Pharmaceuticals, Boston, MA, USA) is a CFTR potentiator, approved for individuals with 96 different gating CFTR mutations as young as 4 months [7]. Approximately 15% of people with CF are eligible for treatment with ivacaftor as monotherapy, based on CFTR mutation. In phase 3 studies of ivacaftor, change in FEV_1_ was greater by 10.6 percent predicted compared to placebo, and these effects were sustained through 48 weeks [8]. Similar effects have been seen in subsequent randomized-control trials and observational studies [9,10]. Given these robust effects, ivacaftor is described as a “highly effective CFTR modulator”.

In class II mutations, including F508Del, ivacaftor is ineffective as monotherapy due to the nature of the underlying gene defect; thus, it is used in combination with a CFTR corrector, either lumacaftor (Orkambi, Vertex Pharmeceuticals) or tezacaftor (Symdeko, Vertex Pharmaceuticals). Given the high prevalence of F508Del mutations, use of dual combination therapy widely expanded the availability of CFTR modulators to the majority of people with CF [5]. Dual combination therapies have led to more modest improvements in pulmonary function, with the mean absolute improvement in FEV_1_ between 2.6 and 4.0 percent predicted with lumacaftor-ivacaftor (LUM-IVA) and 4.0 percent predicted with tezacaftor-ivacaftor (TEZ-IVA) [11,12].

In 2019, the FDA approved the first triple combination therapy, ivacaftor and tezacaftor in combination with elexacaftor, another CFTR corrector (Trikafta, Vertex Pharmaceuticals). Triple combination therapy with elexacaftor, tezacaftor and ivacaftor (ELEX-TEZ-IVA) has proven to be more efficacious than the dual combination therapies in individuals with F508del, with improvements in FEV_1_ of 13.8 percent predicted greater than placebo and 10.0 percent predicted greater than TEZ-IVA [13,14].

Given the superior performance of ELEX-TEZ-IVA as compared to dual combination therapy, it will likely replace LUM-IVA and TEZ-IVA for individuals with F508del mutations, providing a highly effective CFTR modulator for this population. It is currently approved for individuals with CF and at least one F508del who are ≥6 years of age.

In addition to impact on pulmonary function, CFTR modulator therapies also impact growth and nutritional status to varying degrees, again with more robust effects seen with highly effective CFTR modulators (ivacaftor and ELEX-TEZ-IVA) as compared to dual combination therapies (LUM-IVA and TEZ-IVA) [6,9,13,14,15,16,17]. With widespread use of highly effective CFTR modulators, it is necessary to understand not only the full impact of these medications on growth and nutritional status, but also the mechanisms behind these changes. While data for ELEX-TEZ-IVA are currently being collected through ongoing studies, the effects of ivacaftor are better understood, and will be described in this review (Table 1).

## 3. Anthropometrics and Nutritional Status in Cystic Fibrosis

Malnutrition has been a hallmark of CF since the disease was first recognized [18]. Multiple factors related to CFTR dysfunction have been associated with poor nutritional status in people with CF including increased pancreatic dysfunction in the form of exocrine pancreatic insufficiency (EPI or PI), CF-related diabetes, and impaired bicarbonate secretion; increased fatty acid turnover and energy expenditure; and factors associated with enteric homeostasis including bile salt loss in stool, increased inflammation, and small intestinal bacterial overgrowth [19].

Due to these factors, patients with CF are predisposed to undernutrition, including lower weight, height, weight-for-height, and body mass index (BMI) Z-scores than their unaffected peers. Lower nutritional status as measured by these anthropometrics is strongly associated with decreased lung function, increased likelihood of transplant, and decreased survival in patients with CF across the age spectrum [20,21,22,23,24]. Longitudinal studies have revealed that nutritional status in younger years correlates with lung function later in life, and changes in anthropometric z-scores correlate with changes in FEV_1_ measurements [25].

Interventions aimed at improving nutritional status including supplemental nutrition, pancreatic enzyme-replacement therapy, and behavior modification have led to improved nutritional status and lung function [26,27,28]. Implementation of newborn screening and the resulting earlier diagnosis of CF has provided the opportunity for earlier intervention prior to the development of malnutrition and has been credited with improved outcomes and reduced morbidity [29]. However, even with newborn screening, early nutritional intervention, deficits remain in growth and lung function in children with CF compared to age-matched peers [30].

### 3.1. Effects of Ivacaftor on Anthropometrics and Nutritional Status in Cystic Fibrosis

In addition to significant improvements in pulmonary function, patients treated with ivacaftor have demonstrated significant changes in anthropometrics and growth parameters.

The STRIVE study was a phase 3, randomized, double-blind, placebo-controlled trial designed to evaluate the efficacy of ivacaftor in 161 people with CF > 12 years of age with at least one copy of the G551D mutation. After 48 weeks, subjects in the ivacaftor group had gained on average 2.7 kg more weight than placebo [8].

The efficacy of ivacaftor was studied in a younger, healthier population of 6–11 year olds with the G551D mutation through the ENVISION study, a randomized, double-blind, placebo-controlled trial. After 24 weeks of therapy, subjects in the treatment group had gained an average of 1.9 kg more than those in the placebo group. BMI Z-scores improved throughout the study period in the treated group and declined in the placebo group [9]. In a pooled analysis of the ENVISION and STRIVE studies by Borowitz et al., subjects younger than 20 treated with ivacaftor had a greater change in mean weight (4.9 vs. 2.2 kg), weight Z-score (0.29 vs. 0.06), and BMI Z-score (0.26 vs. −0.13) relative to placebo. In subjects older than 20, subjects treated with ivacaftor had a greater mean change in body weight (2.7 vs. −0.2 kg) and BMI (0.9 vs. −0.1 kg/m^2^) relative to placebo. In both age groups, differences between treatment and placebo groups were seen by day 15. There was no correlation between changes in body weight and improvements in pulmonary function [16].

Similar results were seen in longitudinal and observational studies of ivacaftor. In the longitudinal GOAL study, subjects with CF > 6 years of age, with at least one G551D mutation, treated with ivacaftor demonstrated significant increases in BMI (by 0.8 kg/m^2^) and weight (by 2.5 kg) after 6 months of treatment [10]. A post hoc analysis by Stalvey et al. examined growth changes in the 83 subjects ages 6–11 years enrolled in both the GOAL and ENVISION studies. The authors found significant increases in height and weight Z-scores in both studies. Height velocity increased between 3 and 6 months in the GOAL group and was higher than placebo in the ENVISION group [15].

Given the favorable effects of ivacaftor on pulmonary function and growth in children as young as six years, subsequent studies examined its safety, pharmacokinetics, and pharmacodynamics in younger populations. The KIWI study was an open-label study designed to examine these parameters in youth ages 2–5 years with at least one G551D mutation. The two-part study (dose finding and long term safety/pharmacodymanics) investigated secondary outcomes including changes in sweat chloride, body weight, BMI, and height Z-scores. After 24 weeks of ivacaftor, significant increases were seen in weight (0.2) and BMI Z-scores (0.4) in the 33 subjects who completed the treatment part of the study [31].

The ARRIVAL study examined the safety and efficacy of ivacaftor in an even younger population, children aged 12 to 24 months. This phase 3, open-label extension study was designed to examine safety and pharmacokinetics of ivacaftor in this age group and found normal growth parameters for age both at baseline and after 24 weeks of therapy [32].

### 3.2. Effects of Elexacaftor/Tezacaftor/Ivacaftor on Anthropometrics and Nutritional Status in Cystic Fibrosis

In addition to improvements in pulmonary function described in Section 2, subjects treated with ELEX-TEZ-IVA demonstrated significant improvements in anthropometric outcomes [13,14].

In a phase 3, randomized, double-blind, placebo-controlled trial designed to test the safety and efficacy of ELEX-TEZ-IVA in subjects 12 years and older with F508Del CFTR mutations, BMI improved with a mean treatment difference of 1.04 in the treatment group relative to placebo after 24 weeks of treatment [14]. In a phase 3, randomized, double-blind, active-controlled study in which the triple combination therapy was compared to dual combination therapy with TEZ-IVA in subjects homozygous for F508Del mutation, treatment with ELEX-TEZ-IVA resulted in a least squares mean increase in BMI of 0.6 kg/m^2^ and a least squares mean body weight increase of 1.6 kg compared to TEZ-IVA after 4 weeks [13].

Ongoing studies of ELEX-TEZ-IVA include open-label studies to determine the long-term effects and physiologic mechanisms behind these changes. Additionally, studies are being conducted to examine the safety and efficacy of this therapy in younger age groups.

## 4. Body Composition in Cystic Fibrosis

Body composition is abnormal in individuals with CF. In a study by Ionescu et al., dual energy x-ray absorptiometry (DXA) was used to compare fat mass (FM), fat-free mass (FFM), and bone mineral density (BMD) between adults with CF and age-matched healthy controls. Subjects with CF had lower FFM than healthy subjects, while FM was similar. Within the CF group, 30 subjects had normal BMI. Of the 30 subjects with normal BMI, 12 met criteria for “hidden loss” of lean mass, characterized by low FFM as measured by DXA, but BMI within a healthy range. Subjects with hidden loss had lower FEV1, lower sustained maximum inspiratory pressure, higher circulating CRP and more frequent exacerbations than subjects with normal BMI and normal FFM [33].

In a larger study by Sheikh et al., anthropomorphic measurements, body composition and pulmonary function were compared in 208 youth ages 5–21 with pancreatic insufficient CF and a healthy comparison group of 309 subjects without CF. Subjects with CF were found to have lower lean body mass Z-scores (LBM-Z) for BMI-Z than those in the healthy comparison group, and in individuals with CF, both BMI-Z and LBMI-Z were positively associated FEV_1_ predicted [34]. In an observational study of adolescents with CF, Calella et al. also found a significant association between lean body mass as measured by DXA and pulmonary function. There was also a significant but weaker association between BMI and pulmonary function, and no association between total FM and pulmonary function [35].

### Changes in Body Composition with Ivacaftor

Changes in weight with ivacaftor have been accompanied by changes in body composition. In a prospective observational study by Stallings et al. of individuals aged 5–61 years with at least one gating mutation, subjects gained a mean of 2.5 kg and had increases in both fat-free mass (0.9 kg) and fat mass (1.6 kg) after three months of treatment. Changes in weight were positively correlated with percent change in REE (r = 0.46, *p* = 0.028) [36].

A smaller, prospective observational study by Mouzaki et al. examined changes in body composition with ivacaftor in 18 individuals with CF and at least one gating mutation. After 24 months of ivacaftor therapy, significant increases in mean weight and BMI were seen as well as a significant change in fat mass without a significant change in fat-free mass [37].

## 5. Bile Acid Metabolism and Hepatobiliary Disease in Cystic Fibrosis

Bile acid homeostasis is altered in patients with CF, primarily due to impaired reabsorption of bile acids and resulting increased excretion in feces. While the total bile acid pool is equivalent to healthy controls, the composition is significantly different. Under normal conditions, bile acids are synthesized in the liver and secreted via the biliary tree in the duodenum. Ultimately, up to 95% of the luminal bile acids are reabsorbed in the enterocytes of the terminal ileum via the apical sodium-dependent bile acid transporter (ASBT). Reabsorption subsequently downregulates bile acid synthesis in the liver and maintains homeostasis via the nuclear farnesoid X receptor (FXR) pathway: In the enterocyte, reabsorbed bile acids activate FXR, leading to increased expression and release of fibroblast growth factor 19 (FGF19) into the portal circulation. FGF19 then binds to a similar FXR receptor in the hepatocyte nucleus, downregulating bile acid synthesis. Bile acids in the portal circulation also directly bind to hepatic FXR in a secondary negative feedback mechanism [38].

While other mechanisms related to CFTR dysfunction including altered transit time, intestinal pH, and intestinal fat content underlie malnutrition, fecal bile acid loss appears to occur independent of these processes [39]. The thick, dehydrated mucus layer on the intestinal luminal surface that is a result of decreased bicarbonate and fluid secretion directly impedes absorption. Furthermore, dysbiosis associated with CFTR dysfunction likely directly affects luminal bile acid composition via bile acid deconjugation and dihydroxylation, resulting in differential absorption via ASBT or passive absorption [40]. FXR signaling does appear to be impaired in patients with CF, likely reducing negative feedback of hepatic bile acid synthesis. At the same time, biliary secretion is impaired in some patients with CFTR dysfunction due to thicker, more viscous bile as a result of impaired bicarbonate and fluid secretion causing biliary cirrhosis.

Biliary cirrhosis is not the only hepatic complication of cystic fibrosis; up to 25% of patients have some form of CF-related liver disease (CFRLD). Additional clinical manifestations of liver disease include steatosis, gallstones, and liver cancer [41]. Multiple factors may be associated with CFRLD, including CF-related diabetes, malnutrition, and altered bile acid homeostasis. Notably, FXR signaling modulates liver regeneration and proliferation [40]. The progression of CFRLD tends to occur asymptomatically, without typical signs or symptoms of liver dysfunction until later stages. CFRLD correlates with normalization of fecal bile acid excretion, possibly reflecting hepatic synthetic dysfunction [39]. Ursodeoxycholic acid is often used in treatment of CFRLD, though there is insufficient evidence to support any effect on outcomes or disease progression [42].

### Changes in Bile Acid Metabolism and Hepatobiliary Disease with Ivacaftor

Treatment with ivacaftor may result in normalization of enterohepatic circulation. In a combined analysis of subjects with G551D and S1251N gating mutations by Van de Peppel et al., subjects with CF had lower concentrations of FGF-19 and higher 7α-hydroxy-4-cholesten-3-one (C4), a marker of bile acid synthesis, than controls. Treatment with ivacaftor increased FGF-19 and reduced C4 concentrations, both toward normalization [43].

While the effects of highly effective CFTR modulators on CF-related liver disease are largely unknown, significant improvement of hepatic steatosis after treatment with ivacaftor has been reported [44].

## 6. Exocrine Pancreatic Dysfunction in Cystic Fibrosis

Exocrine pancreatic insufficiency (EPI or PI) describes dysfunction of the digestive secretory function of the pancreas and is the principal cause of nutrient malabsorption in patients with CF. The mechanism underlying PI in patients with CF is impaired chloride-dependent bicarbonate and fluid secretion in the pancreatic duct cells with normal acinar cell pancreatic zymogen secretion. The thick pancreatic secretions lead to ductal obstruction, inflammation, fibrosis, and acinar destruction starting in utero [45]. Pancreatic sufficiency (PS) declines with homozygosity and severity of CFTR variant; patients homozygous for class I–III, VI variants with no functional channel are unlikely to have any residual pancreatic function, while patients with at least one milder class IV–V variant are likely to have some degree of pancreatic sufficiency [46]. By 12 months of life, approximately 85% of the population with CF will have PI requiring pancreatic enzyme-replacement therapy (PERT) [47]. Historically, patients with CF and PI have had increased morbidity and decreased life expectancy when compared to their peers with CF who are PS [46]. Multiple consequences of pancreatic acinar destruction and the resulting PI contribute to malabsorption in patients with CF and PI.

First, absence of pancreatic proteases, amylases, and lipases in the gut lumen results in malabsorption of proteins, carbohydrates, and triglycerides, respectively. This contributes to a calorie deficit as well as symptoms of abdominal pain, bloating, and diarrhea. Pancreatic enzyme-replacement therapy is the gold-standard treatment for malabsorption in patients with PI to counteract the paucity of endogenous digestive enzymes. With optimum PERT dosing, the coefficient of fat absorption (CFA) can be improved to 85–95%, where normal for infants is ≥85% and adults is ≥93% [48]. While appropriate PERT prescription can improve studies of dietary fat malabsorption, symptoms of malabsorption or gastrointestinal distress may not resolve entirely. Research has failed to establish clear correlation between PERT dose and outcomes including growth, abdominal pain, gassiness, constipation, or number of stools [49]. The reason underlying this is likely due to a second consequence of PI, decreased intestinal pH due to reduced bicarbonate secretion.

### Changes in Exocrine Pancreatic Dysfunction with Ivacaftor

Pancreatic dysfunction in CF begins in utero, with pancreatic inflammation present at birth. However, emerging evidence suggests that early initiation of ivacaftor may improve pancreatic function in infants and young children with gating mutations.

In the ARRIVAL study, changes in fecal elastase (FE) and immunoreactive trypsinogen (IRT), indirect markers of PI, were measured in the 12–24-month-old subjects at baseline and week 24. The mean absolute change in FE seen among all subjects was +164 μg/g. Of the nine subjects who had low FE at baseline (<50 μg/g), six had improvement to >200 μg/g at week 24. A 56% mean decrease in IRT from baseline, indicating improved pancreatic function, was seen among all study participants at 24 weeks [32].

Similar improvements in markers of pancreatic function were seen in an older population of children ages 2–5 years treated with ivacaftor. In the KIWI study, a mean absolute increase in FE of 99.8 μg/g and a mean decrease in IRT of 20.7 ng/mL were observed [31]. These results were sustained throughout an open-label extension study, the KLIMB study, in which subjects were followed from week 24 (completion of KIWI) to week 84 of ivacaftor therapy. Between weeks 24 and 84, no further significant changes were seen in FE or IRT although trends were seen toward improvement. Of the 18 subjects who had FE measurements at baseline and week 84, one had FE > 200 μg/g at baseline, and five had FE of >200 μg/g at week 84 [50].

In contrast, in an older population (ages 5–61 years), Stallings et al. found no change in FE between baseline and 3 months of ivacaftor therapy. However, the authors did find a significant change in the CFA in subjects with PI, which was positively correlated with the change in FEV1 [36]. Mouzaki et al. evaluated albumin and fat soluble vitamin levels in an older population (mean age 20) after 6 months of ivacaftor, and found no significant change in any of these outcomes [37].

The results of these studies suggest that pancreatic function is more likely to be restored if highly effective CFTR modulator therapies are initiated as early in life as possible. Sun et al. examined this concept in a ferret model by examining the response to ivacaftor when initiated in utero. In ferrets with G551D mutations treated with ivacaftor in utero, there was partial protection from CF-related pancreatic pathology after birth as well as improved glucose tolerance, growth and survival, and reduced mucous accumulation and bacterial infections in the lung. At any postnatal age, withdrawal of ivacaftor reestablished disease [51].

## 7. Bicarbonate Secretion in Cystic Fibrosis

Pancreatic bicarbonate secretion counteracts gastric acid secretion in the lumen of the duodenum and raises the pH of intestinal contents. Lipase requires pH > 4 to remain active, so failure to buffer gastric secretions adequately that leads to decreased lipase activity in the duodenum [52]. To counteract this, most PERT medications have an enteric coating that dissolves at pH > 5. In people with CF and PI, the pH of intestinal contents reaches this threshold more distal than in people without CF, potentially reducing the exposure of digested macronutrients to the absorptive surface of the small bowel [53].

### Changes in Bicarbonate Secretion with Ivacaftor

In a subset of seven subjects enrolled in the GOAL study by Gelfond et al., intestinal pH profiles were examined at baseline and one month after initiation of ivacaftor using a wireless motility capsule. After treatment with ivacaftor, subjects demonstrated a decreased time to reach and sustain pH of 5.5 after gastric emptying (40 min vs. 8 min, *p* = 0.02), which remained significant when PS subjects were removed from the analysis [54].

## 8. Energy Expenditure in Cystic Fibrosis

Resting energy expenditure (REE) is increased in patients with CF compared to values for healthy people [55]. Multiple factors underly the increased energy needs in these patients, though it is likely primarily due to pulmonary and gastrointestinal symptoms of CF. While Stallings et al. found increased REE in adolescent females with CF compared to healthy controls, there was no correlation with FEV1 [56]. In a study of 38 patients with CF ranging in age from 9 to 34 years, Moudiou found a strong correlation between higher REE and PI, but also no correlation with FEV1 [57]. A larger longitudinal study evaluating REE in adolescents validated the findings of these studies, that the elevation in REE was higher in adolescent females than males and correlated strongly with pancreatic insufficiency [58]. As a result, CF organizations worldwide have traditionally recommended that patients with CF exceed expected energy intakes for age, sex, and physical activity.

### Changes in Energy Expenditure with Ivacaftor

Stallings et al. examined REE prior to and after 3 months of ivacaftor therapy, and found that REE decreased by 5.5 ± 12.0% (*p* < 0.05), and total energy expenditure was unchanged. These improvements were greater for subjects with PI than those who were PS. Changes in REE were negatively correlated with change in FEV1 (r = −0.50, *p* = 0.017) [36].

Changes in energy expenditure were also examined by Mouzaki et al., who found a decrease in REE throughout the study period, but in contrast to Stallings et al., these changes in REE did not correlate with individual increases in FEV1 [37].

## 9. Intestinal Microbiota and Inflammation in Cystic Fibrosis

Similar to opportunistic pulmonary infections in patients with CF, the dehydrated, viscous mucus layer on the intestinal epithelial surface provides an altered environment that selects for atypical microbiota. At the same time, patients with cystic fibrosis have elevated markers of intestinal inflammation like fecal calprotectin; efforts have been made to correlate the intestinal microbiota and inflammatory changes in patients with CF [59]. Reductions in diversity of gut bacteria, independent of the degree of pancreatic sufficiency, have been observed early in children with cystic fibrosis. These changes are sustained through the teenage years and reflect alterations in multiple genera including *Clostridium*, *Eubacterium*, *Faecalibacterium*, and *Bacteroides* [60]. For instance, more abundant *Clostridium* have been associated with increased visual findings of intestinal inflammation on video capsule endoscopy [61].

### Changes in Intestinal Microbiota and Inflammation with Ivacaftor

Ooi et al. conducted an observational study to assess the effects of ivacaftor on intestinal microbiota and inflammation before and after a median of 6.1 months of ivacaftor therapy. After treatment with ivacaftor, a significant increase in abundance of *Akkermansia* and decrease in abundance of *Enterobacteriaceae* were observed, consistent with a healthier microbiome. Additionally, there was a decrease in median stool calprotectin, reflecting decreased enteric inflammation (median 154.4 vs. 87.5 *p* = 0.03). Additionally, correlation was seen between favorable changes in microbiota and decreased stool calprotectin concentrations [62].

Stallings et al. also examined fecal calprotectin before and after treatment, finding a decrease in fecal calprotectin of 30 ± 40 μg/g stool (*p* < 0.01) after three months of treatment with ivacaftor [36].

## 10. Future Directions

Highly effective CFTR modulators mark an important advancement in CF therapy and present new clinical questions and challenges. Future studies will examine the effects of highly effective CFTR modulators when started early in life, and long term outcomes in individuals with CF of all ages. Additionally, the current state allows the impact of highly effective CFTR modulators to be judged primarily based on ivacaftor, and future studies will be needed to see if these effects are also found with use of ELEX-TEZ-IVA.

### 10.1. Linear Growth

While weight gain and increased BMI have been demonstrated with highly effective CFTR modulator use, the impact on linear growth has been more modest. As BMI is a calculated value which includes both weight and height, if increases in BMI are due to weight increases alone, this measurement tool may have different clinical significance. Linear growth is an important target of therapeutics in children with CF as maintaining height > 50th percentile is associated with better pulmonary function [63].

The persistence of height deficits in children and adults CF, despite modulator use, may be due to intrinsic bone differences in CF. Supporting the presence of fundamental bone differences in CF, CF mice exhibit genetic inactivation of CFTR in osteoblasts and low bone mass [64]. Early nutritional interventions in infants diagnosed with CF at birth led to normal weight status at age one year, but persistent length deficits [30]. These early length deficits are likely responsible for persistent short stature in youth with CF, as height velocity in mid- to late- childhood was found to be normal [65]. As highly effective CFTR modulators are used in younger patients, it will be important to determine if early initiation will lead to correction of intrinsic bone deficits and allow for appropriate linear growth.

### 10.2. CF Legacy Diet

Current recommendations state that individuals with CF receive > 100% of estimated energy needs for age and sex through a high fat diet [66]. However, given the findings by Stallings et al. which demonstrate decreased energy expenditure after initiation of highly effective modulator therapy, it will be important to determine if these guidelines will continue to be appropriate, or if they will result in excessive weight gain in some patients. Conversely, it is important to consider that many patients with CF remain below target BMI, even after initiation of modulator therapy, and thus may continue to require a more energy dense diet. Thus, the CF diet will require modifications to meet each individual’s unique nutritional needs.

While it is known that BMI below the 50th percentile is associated with worse pulmonary function in CF, it will be important to understand if adverse health outcomes are also associated with higher BMIs. Understanding the impact of overweight and obesity in CF may inform additional guidelines regarding upper thresholds for BMI to promote optimal health. Additionally, in young children, who may have some restoration of pancreatic function after initiation of highly effective CFTR modulator therapy, the macronutrient composition of the CF diet and dosing of pancreatic enzyme-replacement therapy may also need to be revised. Authors should discuss the results and how they can be interpreted from the perspective of previous studies and of the working hypotheses. The findings and their implications should be discussed in the broadest context possible. Future research directions may also be highlighted.

## Figures and Tables

**Table 1:** Summary of the effects of highly effective CFTR modulators on markers of growth, nutritional status and gastrointestinal health.

Outcome	Ivacaftor	ELEX-TEZ-IVA
Weight-Z	Increased	Increased
Height-Z	Increased in youth	To be determined
BMI-Z	Increased	Increased
Fat Mass	Increased	To be determined
Fat-Free Mass	Unchanged to increased	To be determined
Bile Acid Metabolism	Increased FGF-19 and decreased C4	To be determined
CF Liver Disease	To be determined, case reports of improved steatosis	To be determined
Exocrine Pancreatic Insufficiency	Increased fecal elastase at younger ages	To be determined
Bicarbonate Secretion	Decreased time to reach pH 5.5	To be determined
Energy Expenditure	Decreased	To be determined
GI Microbiome	Changes in intestinal flora and decreased calprotectin	To be determined

## Data Availability

For details of the data summarized in this review paper, please contact original authors.

## References

[1] Rowe S.M., Miller S., Sorscher E.J. (2005). Cystic fibrosis. N. Engl. J. Med..

[2] Zeitlin P.L. (2000). Pharmacologic restoration of αδF508 CFTR-mediated chloride current. Kidney Int..

[3] McKone E.F., Emerson S.S., Edwards K.L., Aitken M.L. (2003). Effect of genotype on phenotype and mortality in cystic fibrosis: A retrospective cohort study. Lancet.

[4] Cystic Fibrosis Foundation (2020). Patient Registry 2019 Annual Data Report.

[5] Cuevas-Ocaña S., LaSelva O., Avolio J., Nenna R. (2020). The era of CFTR modulators: Improvements made and remaining challenges. Breathe.

[6] Rowe S.M., Daines C., Ringshausen F.C., Kerem E., Wilson J., Tullis E., Nair N., Simard C., Han L., Ingenito E.P. (2017). Tezacaftor—Ivacaftor in residual-function heterozygotes with cystic fibrosis. N. Engl. J. Med..

[7] Vertex Pharmaceuticals Incorporated (2020). Kalydeco (Ivacaftor) [Package Insert].

[8] Ramsey B.W., Davies J., McElvaney N.G., Tullis E., Bell S.C., Dřevínek P., Griese M., McKone E.F., Wainwright C.E., Konstan M.W. (2011). A CFTR potentiator in patients with cystic fibrosis and the G551DMutation. N. Engl. J. Med..

[9] Davies J.C., Wainwright C., Canny G.J., Chilvers M., Howenstine M.S., Munck A., Mainz J.G., Rodriguez S., Li H., Yen K. (2013). Efficacy and safety of ivacaftor in patients aged 6 to 11 years with cystic fibrosis with a G551D mutation. Am. J. Respir. Crit. Care Med..

[10] Rowe S.M., Heltshe S.L., Gonska T., Donaldson S.H., Borowitz D., Gelfond D., Sagel S.D., Khan U., Mayer-Hamblett N., van Dalfsen J.M. (2014). Clinical mechanism of the cystic fibrosis transmembrane conductance regulator potentiator ivacaftor in G551D-mediated cystic fibrosis. Am. J. Respir. Crit. Care Med..

[11] Wainwright C.E., Elborn J.S., Ramsey B.W., Marigowda G., Huang X., Cipolli M., Colombo C., Davies J.C., de Boeck K., Flume P.A. (2015). Lumacaftor—Ivacaftor in patients with cystic fibrosis homozygous for Phe508del CFTR. N. Engl. J. Med..

[12] Taylor-Cousar J.L., Munck A., McKone E.F., van der Ent C.K., Moeller A., Simard C., Wang L.T., Ingenito E.P., McKee C., Lu Y. (2017). Tezacaftor—Ivacaftor in patients with cystic fibrosis homozygous for Phe508del. N. Engl. J. Med..

[13] Heijerman H.G.M., McKone E.F., Downey D.G., van Braeckel E., Rowe S.M., Tullis E., Mall M.A., Welter J.J., Ramsey B.W., McKee C.M. (2019). Efficacy and safety of the elexacaftor plus tezacaftor plus ivacaftor combination regimen in people with cystic fibrosis homozygous for the F508del mutation: A double-blind, randomised, phase 3 trial. Lancet.

[14] Middleton P.G., Mall M.A., Dřevínek P., Lands L.C., McKone E.F., Polineni D., Ramsey B.W., Taylor-Cousar J.L., Tullis E., Vermeulen F. (2019). Elexacaftor—Tezacaftor—Ivacaftor for cystic fibrosis with a single Phe508del allele. N. Engl. J. Med..

[15] Stalvey M.S., Pace J., Niknian M., Higgins M.N., Tarn V., Davis J., Heltshe S.L., Rowe S.M. (2017). Growth in prepubertal children with cystic fibrosis treated with ivacaftor. Pediatrics.

[16] Borowitz D., Lubarsky B., Wilschanski M., Munck A., Gelfond D., Bodewes F.A.J.A., Schwarzenberg S.J. (2016). Nutritional status improved in cystic fibrosis patients with the G551D mutation after treatment with ivacaftor. Dig. Dis. Sci..

[17] Ratjen F., Hug C., Marigowda G., Tian S., Huang X., Stanojevic S., Milla C.E., Robinson P.D., Waltz D., Davies J.C. (2017). Efficacy and safety of lumacaftor and ivacaftor in patients aged 6–11 years with cystic fibrosis homozygous for F508del-CFTR: A randomised, placebo-controlled phase 3 trial. Lancet Respir. Med..

[18] Singh V.K., Schwarzenberg S.J. (2017). Pancreatic insufficiency in cystic fibrosis. J. Cyst. Fibros..

[19] Brownell J.N., Bashaw H., Stallings V.A. (2019). Growth and nutrition in cystic fibrosis. Semin. Respir. Crit. Care Med..

[20] Kraemer R., Rüdeberg A., Hadorn B., Rossi E. (1978). Relative underweight in cystic fibrosis and its prognostic value. Acta Paediatr..

[21] Richardson I., Nyulasi I., Cameron K., Ball M., Wilson J. (2000). Nutritional status of an adult cystic fibrosis population. Nutrition.

[22] Navarro J., Rainisio M., Harms H., Hodson M., Koch C., Mastella G., Strandvik B., McKenzie S. (2001). Factors associated with poor pulmonary function: Cross-sectional analysis of data from the ERCF. Eur. Respir. J..

[23] Steinkamp G. (2002). Relationship between nutritional status and lung function in cystic fibrosis: Cross sectional and longitudinal analyses from the German CF quality assurance (CFQA) project. Thorax.

[24] Ashkenazi M., Nathan N., Sarouk I., Bar Aluma B.E., Dagan A., Bezalel Y., Keler S., Vilozni D., Efrati O. (2019). Nutritional status in childhood as a prognostic factor in patients with cystic fibrosis. Lung.

[25] Zemel B.S., Jawad A.F., FitzSimmons S., Stallings V.A. (2000). Longitudinal relationship among growth, nutritional status, and pulmonary function in children with cystic fibrosis: Analysis of the Cystic Fibrosis Foundation National CF Patient Registry. J. Pediatr..

[26] Mansell A.L., Andersen J.C., Muttart C.R., Ores C.N., Loeff D.S., Levy J.S., Heird W.C. (1984). Short-term pulmonary effects of total parenteral nutrition in children with cystic fibrosis. J. Pediatr..

[27] Levy L.D., Durie P.R., Pencharz P.B., Corey M.L. (1985). Effects of long-term nutritional rehabilitation on body composition and clinical status in malnourished children and adolescents with cystic fibrosis. J. Pediatr..

[28] Steinkamp G., von der Hardt H. (1994). Improvement of nutritional status and lung function after long-term nocturnal gastrostomy feedings in cystic fibrosis. J. Pediatr..

[29] Siret D., Bretaudeau G., Branger B., Dabadie A., Dagorne M., David V., de Braekeleer M., Moisan-Petit V., Picherot G., Rault G. (2003). Comparing the clinical evolution of cystic fibrosis screened neonatally to that of cystic fibrosis diagnosed from clinical symptoms: A 10-year retrospective study in a French region (Brittany). Pediatr. Pulmonol..

[30] Leung D.H., Heltshe S.L., Borowitz D., Gelfond D., Kloster M., Heubi J.E., Stalvey M., Ramsey B.W. (2017). Effects of diagnosis by newborn screening for cystic fibrosis on weight and length in the first year of life. JAMA Pediatr..

[31] Davies J.C., Cunningham S., Harris W.T., Lapey A., Regelmann W.E., Sawicki G.S., Southern K.W., Robertson S., Green Y., Cooke J. (2016). Safety, pharmacokinetics, and pharmacodynamics of ivacaftor in patients aged 2–5 years with cystic fibrosis and a CFTR gating mutation (KIWI): An open-label, single-arm study. Lancet Respir. Med..

[32] Rosenfeld M., Wainwright C., Higgins M., Wang L.T., McKee C., Campbell D., Tian S., Schneider J., Cunningham S., Davies J.C. (2018). Ivacaftor treatment of cystic fibrosis in children aged 12 to <24 months and with a CFTR gating mutation (ARRIVAL): A phase 3 single-arm study. Lancet Respir. Med..

[33] Ionescu A.A., Evans W.D., Pettit R.J., Nixon L.S., Stone M.D., Shale D.J. (2003). Hidden depletion of fat-free mass and bone mineral density in adults with cystic fibrosis. Chest.

[34] Sheikh S., Zemel B.S., Stallings V.A., Rubenstein R.C., Kelly A. (2014). Body composition and pulmonary function in cystic fibrosis. Front. Pediatr..

[35] Calella P., Valerio G., Thomas M., McCabe H., Taylor J., Brodlie M., Siervo M. (2018). Association between body composition and pulmonary function in children and young people with cystic fibrosis. Nutrition.

[36] Stallings V.A., Sainath N., Oberle M., Bertolaso C., Schall J.I. (2018). Energy balance and mechanisms of weight gain with ivacaftor treatment of cystic fibrosis gating mutations. J. Pediatr..

[37] Kolpen M., Ravnholt C., Qvist T., Kragh K.N., Fritz B.G., Bjarnsholt T., Høiby N., Jensen P. (2017). Poster session abstracts. Pediatr. Pulmonol..

[38] Kim I., Ahn S.-H., Inagaki T., Choi M., Ito S., Guo G.L., Kliewer S.A., Gonzalez F.J. (2007). Differential regulation of bile acid homeostasis by the farnesoid X receptor in liver and intestine. J. Lipid Res..

[39] O’Brien S., Mulcahy H., Fenlon H., O’Broin A., Casey M., Burke A., Fitzgerald M.X., Hegarty J.E. (1993). Intestinal bile acid malabsorption in cystic fibrosis. Gut.

[40] Van de Peppel I.P., Bodewes F.A., Verkade H.J., Jonker J.W. (2019). Bile acid homeostasis in gastrointestinal and metabolic complications of cystic fibrosis. J. Cyst. Fibros..

[41] Feranchak A.P., Sokol R.J. (2001). Cholangiocyte biology and cystic fibrosis liver disease. Semin. Liver Dis..

[42] Cheng K., Ashby D., Smyth R.L. (2017). Ursodeoxycholic acid for cystic fibrosis-related liver disease. Cochrane Database Syst. Rev..

[43] Van de Peppel I., Doktorova M., Berkers G., de Jonge H.R., Houwen R.H., Verkade H.J., Jonker J.W., Bodewes F.A. (2019). IVACAFTOR restores FGF19 regulated bile acid homeostasis in cystic fibrosis patients with an S1251N or a G551D gating mutation. J. Cyst. Fibros..

[44] Hayes D., Warren P.S., McCoy K.S., Sheikh S.I. (2015). Improvement of hepatic steatosis in cystic fibrosis with ivacaftor therapy. J. Pediatr. Gastroenterol. Nutr..

[45] Durie P.R., Forstner G.G. (1989). Pathophysiology of the exocrine pancreas in cystic fibrosis. J. R. Soc. Med..

[46] Wilschanski M., Durie P.R. (1998). Pathology of pancreatic and intestinal disorders in cystic fibrosis. J. R. Soc. Med..

[47] Kerem E., Corey M., Kerem B.-S., Rommens J., Markiewicz D., Levison H., Tsui L.-C., Durie P. (1990). The relation between genotype and phenotype in cystic fibrosis—Analysis of the most common mutation (ΔF508). N. Engl. J. Med..

[48] Littlewood J.M., Wolfe S.P., Conway S.P. (2006). Diagnosis and treatment of intestinal malabsorption in cystic fibrosis. Pediatr. Pulmonol..

[49] Baker S.S., Borowitz D., Duffy L., Fitzpatrick L., Gyamfi J., Baker R.D. (2005). Pancreatic enzyme therapy and clinical outcomes in patients with cystic fibrosis. J. Pediatr..

[50] Rosenfeld M., Cunningham S., Harris W.T., Lapey A., Regelmann W.E., Sawicki G.S., Southern K.W., Chilvers M., Higgins M., Tian S. (2019). An open-label extension study of ivacaftor in children with CF and a CFTR gating mutation initiating treatment at age 2–5 years (KLIMB). J. Cyst. Fibros..

[51] Sun X., Yi Y., Yan Z., Rosen B.H., Liang B., Winter M.C., Evans T.I.A., Rotti P.G., Yang Y., Gray J. (2019). In utero and postnatal VX-770 administration rescues multiorgan disease in a ferret model of cystic fibrosis. Sci. Transl. Med..

[52] Trang T., Chan J., Graham D.Y. (2014). Pancreatic enzyme replacement therapy for pancreatic exocrine insufficiency in the 21st century. World J. Gastroenterol..

[53] Delchier J., Vidon N., Girardin M.-F.S.-M., Soule J.-C., Moulin C., Huchet B., Zylberberg P. (2007). Fate of orally ingested enzymes in pancreatic insufficiency: Comparison of two pancreatic enzyme preparations. Aliment. Pharmacol. Ther..

[54] Gelfond D., Heltshe S., Ma C., Rowe S.M., Frederick C., Uluer A., Sicilian L., Konstan M., Tullis E., Roach C.R.N. (2017). Impact of CFTR modulation on intestinal pH, motility, and clinical outcomes in patients with cystic fibrosis and the G551D mutation. Clin. Transl. Gastroenterol..

[55] Vaisman N., Pencharz P.B., Corey M., Canny G.J., Hahn E. (1987). Energy expenditure of patients with cystic fibrosis. J. Pediatr..

[56] Stallings V.A., Tomezsko J.L., Schall J.I., Mascarenhas M.R., Stettler N., Scanlin T.F., Zemel B.S. (2005). Adolescent development and energy expenditure in females with cystic fibrosis. Clin. Nutr..

[57] Moudiou T., Galli-Tsinopoulou A., Nousia-Arvanitakis S. (2007). Effect of exocrine pancreatic function on resting energy expenditure in cystic fibrosis. Acta Paediatr..

[58] Magoffin A., Allen J.R., McCauley J., Gruca M.A., Peat J., van Asperen P., Gaskin K. (2008). Longitudinal analysis of resting energy expenditure in patients with cystic fibrosis. J. Pediatr..

[59] Lee J.M., Leach S., Katz T., Day A.S., Jaffe A., Ooi C. (2012). Update of faecal markers of inflammation in children with cystic fibrosis. Mediat. Inflamm..

[60] Nielsen S., Needham B., Leach S.T., Day A.S., Jaffe A., Thomas T., Ooi C. (2016). Disrupted progression of the intestinal microbiota with age in children with cystic fibrosis. Sci. Rep..

[61] Flass T., Tong S., Frank D.N., Wagner B., Robertson C., Kotter C.V., Sokol R.J., Zemanick E., Accurso F., Hoffenberg E. (2015). Intestinal lesions are associated with altered intestinal microbiome and are more frequent in children and young adults with cystic fibrosis and cirrhosis. PLoS ONE.

[62] Ooi C.Y., Syed S.A., Rossi L., Garg M., Needham B., Avolio J., Young K., Surette M.G., Gonska T. (2018). Impact of CFTR modulation with ivacaftor on gut microbiota and intestinal inflammation. Sci. Rep..

[63] Sanders D.B., Slaven J.E., Maguiness K., Chmiel J.F., Ren C.L. (2021). Early life height attainment in cystic fibrosis is associated with pulmonary function at age 6 years. Ann. Am. Thorac. Soc..

[64] Stalvey M.S., Clines K.L., Havasi V., McKibbin C.R., Dunn L.K., Chung W.J., Clines G.A. (2013). Osteoblast CFTR inactivation reduces differentiation and osteoprotegerin expression in a mouse model of cystic fibrosis-related bone disease. PLoS ONE.

[65] Zysman-Colman Z.N., Kilberg M.J., Harrison V.S., Chesi A., Grant S.F.A., Mitchell J., Sheikh S., Hadjiliadis D., Rickels M.R., Rubenstein R.C. (2021). Genetic potential and height velocity during childhood and adolescence do not fully account for shorter stature in cystic fibrosis. Pediatr. Res..

[66] Turck D., Braegger C.P., Colombo C., Declercq D., Morton A., Pancheva R., Robberecht E., Stern M., Strandvik B., Wolfe S. (2016). ESPEN-ESPGHAN-ECFS guidelines on nutrition care for infants, children, and adults with cystic fibrosis. Clin. Nutr..

